# Blockchain for the Internet of Vehicles: A Decentralized IoT Solution for Vehicles Communication Using Ethereum

**DOI:** 10.3390/s20143928

**Published:** 2020-07-15

**Authors:** Rateb Jabbar, Mohamed Kharbeche, Khalifa Al-Khalifa, Moez Krichen, Kamel Barkaoui

**Affiliations:** 1 Qatar Transportation and Traffic Safety Center, College of Engineering, Qatar University, Doha P.O. Box 2713, Qatar; mkharbec@qu.edu.qa; 2 Cedric Lab, Computer Science Department, Conservatoire National des Arts et Métiers, 75141 Paris, France; kamel.barkaoui@cnam.fr; 3 College of the North Atlantic-QAtar, Doha P.O. Box 24449, Qatar; alkhalifa@cna-qatar.edu.qa; 4 ReDCAD Laboratory, National School of Engineers of Sfax, University of Sfax, Sfax 3038, Tunisia; moez.krichen@redcad.org

**Keywords:** blockchain, automotive communication, internet of vehicles, intelligent transport system, internet of things, ethereum, security, cloud and android

## Abstract

The concept of smart cities has become prominent in modern metropolises due to the emergence of embedded and connected smart devices, systems, and technologies. They have enabled the connection of every “thing” to the Internet. Therefore, in the upcoming era of the Internet of Things, the Internet of Vehicles (IoV) will play a crucial role in newly developed smart cities. The IoV has the potential to solve various traffic and road safety problems effectively in order to prevent fatal crashes. However, a particular challenge in the IoV, especially in Vehicle-to-Vehicle (V2V) and Vehicle-to-Infrastructure (V2I) communications, is to ensure fast, secure transmission and accurate recording of the data. In order to overcome these challenges, this work is adapting Blockchain technology for real time application (RTA) to solve Vehicle-to-Everything (V2X) communications problems. Therefore, the main novelty of this paper is to develop a Blockchain-based IoT system in order to establish secure communication and create an entirely decentralized cloud computing platform. Moreover, the authors qualitatively tested the performance and resilience of the proposed system against common security attacks. Computational tests showed that the proposed solution solved the main challenges of Vehicle-to-X (V2X) communications such as security, centralization, and lack of privacy. In addition, it guaranteed an easy data exchange between different actors of intelligent transportation systems.

## 1. Introduction

The Internet of things (IoT) represents a network of physical devices embedded with network connectivity, actuators, sensors, software and electronics, such as home appliances, vehicles, and other items. Through the embedded computing systems, each device is identified and incorporated into the Internet infrastructure. The network infrastructure allows remote control of such devices [1]. Accordingly, computer-based systems are increasingly integrated into the physical world, which leads to economic gains, accuracy, and efficiency, and decreased human intervention. Thanks to actuators and sensors, the technology represents the general class of cyber-physical systems, including smart cities, intelligent transportation, smart homes, virtual power plants, and smart grids.

The IoT is transforming conventional vehicular ad-hoc networks (VANETs) into the Internet of Vehicles (IoV) [2]. The IoV represents the real time data interaction between vehicles and between vehicles and infrastructures through smart terminal devices, vehicle navigation systems, mobile communication technology and information platforms that allow information interaction and share driving instructions, and control the network system.

This concept has enabled easier collection and sharing of information about vehicles and infrastructures. It also allows data collection, computing and sharing into Internet systems and other information platforms.

Recently, this concept has taken firm ground in reality. It is estimated that in the near future, 25 billion things will be connected to the Internet, and vehicles will account for a significant number [2]. So far, Intelligent Transportation Systems (ITS) in Japan and Europe have already implemented some forms of IoV technology, while 55,000 licensed rickshaws in New Delhi have been equipped with GPS devices [3]. Due to the prompt development of communication and computation technologies, this concept has attracted enormous research and commercial interest. What is more, smart vehicles are increasingly connected to the Internet, other vehicles nearby, and traffic management systems. In this way, vehicles are being incorporated into the Internet of Things (IoT). Nevertheless, in spite of indubitable advantages, this concept has certain limitations. Importantly, due to their high connectivity, it is really hard to ensure the security of smart vehicles, making them susceptible to malicious entities. Moreover, a sensitive data exchange leads to additional privacy challenges. The conventional security and privacy methods are ineffective when it comes to smart vehicles. The main challenges are summarized as follows:

- Centralization: At the moment, smart vehicle architectures are based on centralized, brokered communication models [4]. More precisely, central cloud servers identify, authenticate, authorize, and connect all the vehicles. Nevertheless, it is not likely that this model will be scaled. The failure of cloud servers can endanger the whole network.
- Lack of Privacy: Typically, user privacy is not protected in the current communication architectures. In other words, data pertaining to the vehicle is exchanged without the owner’s permission. Moreover, noisy or summarized data is revealed to the requester.
- Heterogeneity: The use of connecting devices in IoV is highly variable, as they are deployed by different entities, authorities, and individuals. Moreover, their resolutions, functionalities, and operating conditions differ from each other. Hence, it is challenging to enable the smooth integration of numerous devices at the same time. In particular, merging such devices in a complex network increases the degree of complexity.
- Scalability: A use of miniaturized devices such as actuators and sensors has been increasing due to the prompt rise in embedded technologies. Simultaneously, the data created by such devices is growing indefinitely. Thus, another significant challenge related to the IoV is to manage the number of devices and the data they create.
- Interoperability: Both human and non-human objects represent actors in the IoV ecosystem. Each actor, depending on the environment and the particular situation, can play several roles, such as service providers, data consumer, data provider, and available resource in IoV applications. To materialize the vision of the IoV, it is essential to ensure the smooth interaction of all the actors. If each actor is managed in a different way, their interaction magnifies.
- Mobility: The challenges in terms of mobility are related to protocol efficiency and the IoT network. Currently, the use of sensor networks, Mobile Ad Hoc Networks (MANETs), and mobility protocols of Vehicular Ad Hoc Networks (VANETs) are not adequately equipped to handle standard IoT device because of considerable processing and energy constraints. Moreover, efficient real-time authentication is required instead of the one-time initial configuration considering that the vehicle must continuously authenticate other vehicles present on the roads.
- Safety Threats: The number of autonomous driving functions in smart vehicles keeps growing. Consequently, a security breach that occurs occurred by a malfunction resulting from the installation of malicious software can lead to car crashes and endanger road users.

VANETs are susceptible to attacks such as Man-In-The-Middle (MITM) attacks and thus, it is very challenging to ensure its security [5,6,7]. Malicious node (MITM) uses these attacks to alter or eavesdrop messages exchanged between vehicles containing delay-intolerant or sensitive information, e.g., a steep-curve warning. Consequently, incorrect and compromised information can be spread throughout the network. What is more, Distributed Denial of Service (DDOS) can be used to block users from accessing network services. In addition, DDOS in the environment of the connected vehicle can manipulate identities and disseminate fake information to consequently introduce a jam in the targeted network [8]. Accordingly, the main principles of the security requirement integrity, confidentiality, and availability, are violated. In this context, Troy Hunt, a Microsoft Most Valuable Professional (MVP) in Security, gained access in February 2016 to vehicle data and controlled certain vehicle systems [9] from his home in Australia. Then, in June 2016, a Mitsubishi Outlander PHEV [10] was hacked via its remote control smartphone app, which allowed the user to lock or unlock doors, edit charger settings and control non-driving critical elements such as air conditioning. Tesla [11] has fallen victim to several hacks, most recently at the hands of Chinese cybersecurity firm Keen Security Lab. In addition, researchers [12] were able to control the brakes, doors, and mirrors of a Tesla Model S from 20 km (12 m) away. All these incidents indicate the serious need for an advanced solution for communication and data transmission providing integrity, security and efficiency. Recently, Blockchain [13,14] technology has shown a potential to improve the intelligent transport systems by making them secure, distributed, and autonomous. In this way, the infrastructure and resources of intelligent transport systems such as crowd sourcing technology can be used more effectively. Blockchain is crucial for addressing the challenges of privacy and security on the IoV networks. To overcome these challenges, a Decentralized IoT Solution for Vehicles communication (DISV) based on the concept of Blockchain is proposed. More precisely, every member of the Internet of Vehicle networks receives messages and subsequently broadcasts them to selected Blockchain layer. Furthermore, the server will verify the received block based on its local knowledge and make a decision whether it should be added to the smart contract. In this paper, the main contributions are three-fold as follows:

- A new IoT Solution (DISV) to study all the possible interactions between different components and road users of smart cities, such vehicles, lights, radars, pedestrians and others. This DISV consists of three primary layers:
  - The perception layer that consists of multiple Android applications (AV and AP) which are designed to sense and collect information about vehicles, drivers, and infrastructures.
  - The network layer, which enables data transfer between devices and the cloud through networks such as wireless or 4G.
  - The application layer, which consists of cloud solutions responsible for management, data analysis, and providing services to the user.
- A Decentralized Framework based on Blockchain technology with real time application (RTA) specification aimed at enabling secure communication between vehicles and other actors in transportation systems.
- Good performance of the proposed system particularly in terms of the execution time, costs, availability, integrity, immutability, and security.

The remainder of this paper is organized as follows. In Section 2, an overview of Blockchain techniques and Ethereum is presented. Section 3 reviews the Blockchain techniques for the Internet of Things and practically for the Internet of Vehicles. The detailed design of the proposed system is introduced in Section 4 including the components and the main procedures. In Section 5, the results of the computational tests to verify the effectiveness and reliability of the proposed system are described. Section 6 summarizes the main finding and gives new directions for future research.

## 2. Blockchain Technique and Ethereum

Bitcoin, a decentralized global currency cryptosystem, was introduced in 2008 by Satoshi Nakamoto [15], based on the Blockchain technology. In Bitcoin, Blockchain is used to ensure secure exchange of digital money for goods and services without a central authority through a trusted peer-to-peer network in a pseudo-anonymous way. Blockchain or public ledger contains a record of all transactions, accessible by all participants in the network, as they are publicly announced. More precisely, each participant possesses the exact copy of the Blockchain. Individual transactions are separated into blocks, which are time-stamped and published. Subsequently, transactions cannot be modified or reversed, as the hash of the previous blocks is included in the next successors in each block of the chain. Accordingly, there is a consensus about the participants about the history of transactions. Following its successful use in bitcoin, Blockchain as a distributed ledger technology or, more precisely, a distributed database of records, has gained the wide application not only in the current digital economy, but in non-financial fields as well. Primarily, it has become a reliable infrastructure for creating platforms that provide various solutions. The Blockchain technology has become a basis of platforms used in healthcare, transportation, payment processing and money transfers, supply chains, etc., [16]. Still, the most popular use of Blockchain is the development of alternative cryptocurrencies, improved in comparison to Bitcoin in regard to hashing algorithms and proof-of-work algorithm. These improvements are primarily aimed at shortening the verification time of transactions. Since the emergence of Bitcoin, the number of digital currencies has reached 3,000. Currently, the market cap is estimated at $295 billion USD. In addition to Bitcoin, currencies that have gained the most prominence are Ethereum [17] , Ripple [18], and Stellar [19]. The current study is using the Ethereum platform, which will be discussed in detail in subsequent sections.

### 2.1. Ethereum

Ethereum, developed by Vitalik Buterin [20] in 2013 and launched in 2015 following a successful online crowd sale, is a distributed computing platform based on Blockchain. Moreover, it is programmable Blockchain, which allows the users to create new applications as Ethereum runs the programming code of decentralized applications . More specifically, a programming code is called a smart contract, and it allows exchange of money, data, and content by generating, deploying, and operating Decentralized Software applications based on the Blockchain technology.

#### 2.1.1. Ethereum Virtual Machine (EVM)

The Ethereum Virtual Machine (EVM) is a Turing complete software that enables the users to deploy and run different programs in various programming languages (i.e., Solidity) and significantly facilitates the creation of Blockchain applications. As a sandbox environment, it is isolated from the rest of the main network. Thus, each Ethereum network operates its own EVM implementation by executing the same instructions. Computations in the EVM are paid by the currency of Ethereum—Ether (ETH).

#### 2.1.2. Transactions

A term transaction refers to a signed data package and consists of messages sent between Ethereum accounts (the transaction sender to the recipient of the transaction). The transactions are verified through the mining process, or more precisely generating a signature by the private key owned by a transaction sender. The transaction includes the Ethereum recipient address, ETH gasPrice (the cost of a unit of gas consumed), the transferred amount (the maximum amount of gas to be used), and other optional data.

#### 2.1.3. Ether and Gas

Ether is a volatile currency of Ethereum paid by the transaction sender for executing the code in a contract triggered by a message or a transaction and storage and computation. Furthermore, the execution cost or the cost of network use is expressed in gas. More precisely, gas is a unit which expresses the amount of computation effort by minors to execute a given operation, and it is fixed. Thus, the users use ETH to pay the amount of gas needed for the execution. The miners represent the Ethereum network nodes that receive, propagate, verify, and execute transactions. As different operations require different amounts of gas, the gas limit is the maximum amount of gas that the sender will pay for the transactions. More details about Ether and Gas can be found in [21].

#### 2.1.4. Proof-of-Work (PoW)

Proof-of-Work represents the original consensus algorithm that determines the validity of the transaction used in Blockchain to ensure security. Through PoW, miners solve mathematical problems by using special software. Accordingly, miners form the blocks of transactions and add them to Ethereum by connected them to the previous block. The hash of the previous block is incorporated into every new block. Thus, the added block becomes a part of the chain that can be traced until the first or so-called genesis block.

## 3. Literature Review

Blockchain technology is used in almost all domains of the IoT. In the domain of healthcare, the IoT enables feeding the E-healthcare systems with clinical data about patients as well as their healthcare providers, families, and social circles. The healthcare providers store data in the form of Electronic Medical Records (EMRs). The patient data portability of Electronic Health Records EHRs is enhanced in comparison to EMRs. Esposito et al. [22] used the concept of the distributed online database to create a scheme established on Blockchain for using the IoT in the sector of healthcare. Accordingly, Liang et al. [23] employed the Blockchain network in Android healthcare apps for investigation, inspection of records, and protection of integrity. In the Telecommunication, 5G will enhance the IoT by enabling a completely connected and mobile society through the connection of billions of objects [24]. However, the 5G heterogeneous communication environment will have a challenge of protecting privacy. To resolve this issue, Fan et al. [25] developed a scheme using Blockchain aimed at sharing data and ensuring privacy.

In the Internet of Cloud (IoC), data from the millions of IoT devices are uploaded through the Cloud by the Internet connection on the basis of virtualization technology. Xu et al. [26] developed a Blockchain-based intelligent resource management for cloud data centers. Recently, The Internet of Vehicles (IoV) is highly innovative, and there is a new trend of many applications in this field of research. It is based on the integration of vehicles into the IoT with an aim of establishing smart communication between vehicles and different types of networks such as vehicle-to-sensor, vehicle-to-human, vehicle-to-road and vehicle-to vehicle. In many applications, the security model is not centralized. For that reason, Huang et al. [27] developed an ecosystem model on the basis of Blockchain electric vehicle and charging pile management. This model employs Elliptic Curve Cryptography (ECC) for the computation of hash functions of charging piles of electric vehicles. Furthermore, Kang et al. [28] developed PETCON, a P2P electricity-trading system, for illustrating localized and comprehensive operations of P2P electricity trading. The PETCON system employs a consortium Blockchain method to analyze, verify, and share transaction records publicly, while it is not necessary to have a reliable authority. Besides, CreditCoin, a privacy-preserving scheme, was created by Li et al. [29] in order to ensure that adequate announcements are forwarded without revealing users’ identities. This scheme employs the Blockchain for sending anonymous announcements through an aggregation protocol between vehicles. As a result, confidence in IoV information sharing is enhanced. Finally, Yang et al. [30] proposed a Blockchain-based reputation system to assess data credibility in the IoV. Depending on the senders’reputation values, this system judges if the received messages are true or false. Yong, Yuan, et al. [14] developed a Blockchain solution aimed at solving security problems and performance limitations in an Intelligent Transportation Systems (ITS).

The authors combined mandatory information of vehicles (i.e., vehicle insurance, vehicle tax, and traffic regulations) with other relevant information such as the provision of updates and information on weather forecasts and traffic jams.

The main goal is to connect VANET services and use the multiple functionalities of Blockchain including peer-to-peer communication without disclosing personal information, security, and communication between vehicles. Lei et al. [31] introduced dynamic key management using Blockchain for establishing communication systems to be used in vehicles that do not need the administration from the central manager. By relying on a decentralized Blockchain structure, it eliminates any other authorities. Thus, the key transfer process is the process of verification and authentication verified and authenticated by the security manager network.

Dorri et al. [4] created a Blockchain technology mechanism that does not reveal any private information of vehicle users. Simultaneously, it updates the wireless remote software and other emerging vehicle services (i.e., electric vehicles and smart charging services, insurance and car sharing services).

One of the challenges facing the field of the IoV is latency, which typically takes place when real-time cloud services are provided to vehicular cloud subscribers, considering that all models aimed at improving the security and quality of the system require additional communication procedures. Fog computing can be used for delivering real-time services to vehicular cloud subscribers to minimize latency [32]. The proposed solution utilized Networked Fog Centers (NetFCs) at the edge of the network and employed single-hop mobile links (i.e. I2V TCP/IP-based) to connect them to vehicular clouds. Consequently, vehicular service delivery time is minimized, whereas the overall efficiency is maximized. The authors of [33] developed an adaptive resource management controller for Vehicular-to-Infrastructure emerging non-safety services. Their focus was on traffic offloading to local/remote clouds in V2I applications. Such applications download and upload enormous amounts of data and need QoS (Quality of Service). According to the results, hard reliability of the controller to cloud service providers on a per-slot basis in ensured. The innovative solution [34] developed by Aloqaily et al. incorporated the concept of Smart Vehicle as a Service (SVaaS) aimed at ensuring continuous vehicular services in smart cities. The solution detects the future location of the vehicle by using a location prediction mechanism. Following the determination of the predicted location of a vehicle, this solution applies a service selection mechanism based on Quality of Experience (QoE) to select the required services before the vehicle reaches the destination.

In [35], Al Ridhawi al., developed the concept of service availability further to encompass diversified vehicular cloud services. These services were obtained through content sharing by cloud service users and providers. Cutting-edge cloud negotiation entity and a future location prediction model were used to identify and chose the service in local and global cloud service areas.

Due to the importance of vehicle-to-everything’ communication in smart cities and in order to enhance and secure the communications, a new IoT Blockchain-based system was developed and tested. The proposed system has shown to establish secure communications and create entirely decentralized cloud computing for the IoV. In the following section, more details are presented about the proposed solution.

Regarding the V2V communications, many researchers and industry stakeholders have focused on the development of intelligent communication concepts such as smart cities, electric vehicles, Blockchain-based automotive communications approaches, Intelligent Transportation Systems, etc., Table 1 presents a detailed comparison between existing literature on on the Blockchain solution for vehicle communication and proposed work. To begin with, M. Singh et al. [36,37,38] contributed to reinforcing vehicle-to-vehicle data sharing security by proposing a novel Intelligent Vehicle-Trust Point (IV-TP) to ensure secure and fast vehicular communication. This approach is an intelligent data transmission system based on blockchain technology, smart connected vehicles, and vehicular cloud computing. IV-TP assigns unique identity to each smart vehicle, which enables the vehicle to be recognized during a communication session. In particular, an important contribution of IV-TP is that it guarantees the trustworthiness of such vehicle. Furthermore, J. Kang et al. [39] proposed a consortium blockchain-based distributed data management system for VECONs. Roadside Units (RSUs) use smart contracts to verify and store securely data as well the sharing data history. In addition, they consider time of events, paths resemblance and frequency of interaction to build a repute share data system. However, the central architecture of traditional VANETs possesses certain limits and security vulnerabilities . To overcome these limits, B. Leidin et al. [40] proposed an automatic management, communication, and organization framework for distributed vehicular network that relies on Ethereum. Moreover, E. Reilly et al. [41] implemented an innovative light client protocol where communication integrity is ensured within smart cities and related IoT devices. The novelty introduced by the authors is an obligatory an authentication phase of data provenance run on an Ethereum address, which ensured that a transaction session is trusted. In addition, the costs of the transaction through the proposed protocol is lower than through the Bitcoin-based NeuroMesh protocol. However, certain limitations restrict the use of this application in some cases. In fact, as an operating system is needed, not all devices can support it. Besides, the solution is not suitable for time sensitive applications because transactions can be subject of delays. Updating vehicular automotive systems are sensitive to malicious interventions or attacks as well. In order to make this phase safer, G. Falco et al. [42] designed an automotive mechanism that validates sources of vehicular data and guarantees reliability, immutability, and integrity of shared data. The proposed approach uses efficiently the limited computing and networking resources of vehicular and ensures network scalability. Data changes such as software updates, cars identification, and distance travelled are validated through a DHTs-based consensus voting system. Blockchain technology is also employed to share data share in a trustworthy way. Finally, S. Rowan et al. [43] exploited both physical ultrasonic audio and visual light channels to establish V2V communication as a robust and secure way of data sharing. First, a verification phase is conducted to identify the ID and the position of the automobile in order to establish communication. Once verified, the correspondent ID is accepted for further exchange of data. The authors also proposed a novel protocol for key production used for inter-exchange vehicles session, based on a public key Blockchain architecture with the use of physical channels already mentioned. It is important to mention that the previous works have many limitations such as support Turing-Completeness. More specifically, most of the previous works use bitcoin as a Blockchain implementation platform. However, since bitcoin is primarily a cryptocurrency for buying and selling commodities in a safe marketplace pseudo- anonymously, it does not accept smart contract and neither it includes programming features for solving computation problems in order to allow the transfer of different sensitive information. To establish cutting-edge vehicles communication applications, the underlying Blockchain platform must support Turing-complete operations such as Ethereum. In addition, the previous works did not include the performance calculation, in particular execution time, considering that the Blockchain is based on mining processing. More precisely, the data must be mined in order to be shared in the Blockchain. The duration of the mining process can reach up to 10 min [44]. The timing depends on technologies, architectures and type of smart contract and data used. Therefore, it may cannot support real time warning such as alerting driver for potential crashes. Hence, the efficacy of any proposed Blockchain for vehicles communication is limited due to the time-consuming nature of the mining process. The proposed solution in this work is a Decentralized IoT Solution for Vehicles communication (DISV) based on the concept of Blockchain, with the real-time aspect that supports Turing-Completeness as it is developed using Ethereum platform. The performance test represents the proof of the real-time aspect of the developed solution.

## 4. Proposed Solution

### 4.1. System Overview

The purpose of this paper is to present an IoV solution with Real-Time Application (RTA). This solution provides secure communication between vehicles and other actors in transportation systems. It attempts to overcome limitations such as execution time and accordingly, improves performance. A prototype of DISV was developed and tested it based on the following scenario: if a driver is drowsy, the nearest cars should be alerted by sending a message via Blockchain. Since it is based on an IoT architecture, the proposed solution should contain mainly three layers; the perception, the network, and the application layers, as illustrated in Table 2 and described below:

1. The perception layer is the physical layer. It consists of several IoT devices equipped with sensors designed to identify and collect information about the environment (i.e., physical parameters) and to detect nearby smart objects. The Android Application for Vehicles (AV) embedded into the perception layer collects and analyze data about the trip, the vehicle, and the driver’s behavior. Android Application for Infrastructure (AP) simulates the role of IoT devices integrated into the roads such as radars, traffic lights, roadside electronic signs and others.
2. The network layer connects the sensors to other servers, network devices, and smart things, and also transmits and processes sensor data.
3. The application layer consists of Blockchain application and Central Cloud Server. It delivers application-specific services to the IoT devices. More precisely, the Blockchain application manages communication between vehicles and other actors in the transportation system. The Central Cloud Server is in charge of processing and analyzing the obtained data and managing invitations of other actors.

**Figure 1:**
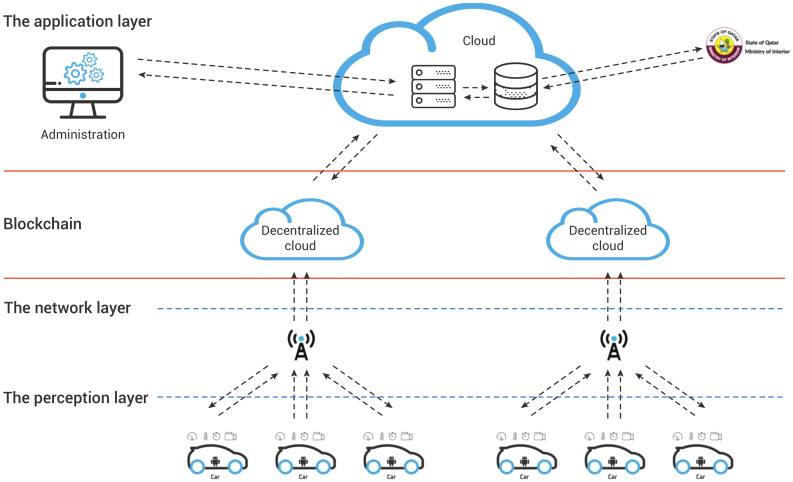
The architecture of the proposed Internet of Things solution.

The Figure 1 depicts the architecture of the proposed solution and demonstrates the principal workflow, which contains three main steps. First, the cars send data to the central server (1). Second, based on the received data, the central server sends an invitation to connect to the Blockchain layer (2). Third, the cars can share the data with the others participants of the IoV in the same area securely (3).

### 4.2. The Perception Layer

In order to test possible scenarios involving various components, an Android applications has been developed in the Android Application for Vehicles (AV) and for infrastructure (AP) as detailed in the following sections.

#### 4.2.1. Android Application for Vehicles (AV)

AV is an Android application consisting of two sub-systems. The first subsystem is the Vehicle Data Collection System (VDCS) which collects data about the trip and the car. The second one is the Driver Drowsiness Detection system that collects data about the driver’s behavior to identify if drowsy or not

**Figure 2:**
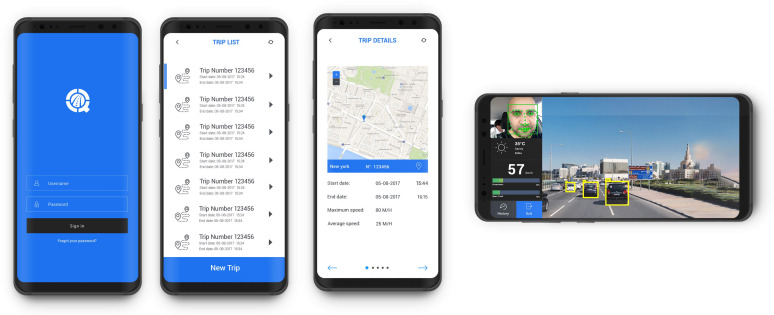
Screenshot of the four main pages of the Android application for Vehicles (AV).

1. VDCS is designed to collect information about the car, such as the car model and characteristics of the motor including horsepower, speed and engine size. Finally, the system collects the data related to the trip such as start and end time, distance, and minimum, maximum, and average speed as illustrated in Figure 2. It is set to detect measures such as rotational velocity along the Roll, Pitch and Yaw axes; acceleration; distance; and GPS position every 15 s.
2. The purpose of Driver Drowsiness Detection is to detect driver’s drowsiness and prevent potential accidents it might cause. This system is an element of the Advanced Driver Assistance System (ADAS), which is an integral part of contemporary automotive technology. The role of ADAS is to improve safety and ensure the satisfying driving experience. This system was developed on the basis of Real-Time Driver Drowsiness Detection using Deep Neural Networks techniques. More details about this system can be found in [45,46,47] .

Mainly, the Android application has four pages. The first page serves for logging in by using a username and password. Following the authentication, the user can start a new trip or access the information about the last five trips in the second page.

If the user chooses a new trip, the application will start recording and displaying all information as described in the previous section. Then, it will send the collected data via the web service to the cloud server. In the fourth page, the front camera will capture and display the driver’s face.

#### 4.2.2. Android Application for Infrastructure (AP)

The aim of this application is to simulate the role of IoT devices integrated into the roads such as radars, traffic lights, roadside electronic signs and others.

Many additional options of the Android application, such as traffic jams, the speed of cars, and weather conditions can be added to the perception layer.

### 4.3. The Network Layer

The network layer establishes the connection between the servers and transmits, and processes the sensor data. The application can use either Wi-Fi or mobile internet (3G/3G+/4G) to send the data to the server.

This collection process uses the hybrid system to gather and store data locally before transmitting it them to the server. This technique is proven to be highly effective for data collection when the Internet connection is poor or unstable.

### 4.4. The Application Layer

Regarding the application layer, it contains two principal compounds: Central cloud server and the communication system using a Blockchain Network.

#### 4.4.1. Central Cloud Server

The central cloud delivers application-specific services to the end-user. It sends the collected data to the web services for processing and analysis before showing them to the end-user. The web service is a component of the application layer responsible for interaction between different components of the IoT solution, such as web site, database serve, IoT devices, and embedded systems. Windows Communication Foundation by Microsoft is used to implement the web service based on the REST Architecture and JSON message format. In addition to collecting data from devices, it uses information about crashes from the General Directorate of Traffic at the Ministry of Interior as well as road conditions or any relevant data from other authorities. The data is available to the end-user through the website with direct access to the web services.

**Figure 3:**
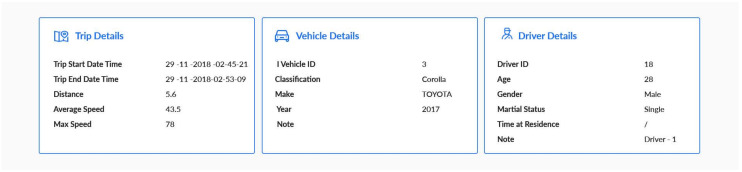
Screenshot of a real trip displaying information about the vehicle and the driver.

The web application is the interface the researchers use to interact and query the recorded data. The website content, shown in Figure 3, displays demographic information about the driver’s nationality, gender and age. It also includes information about the vehicle, including the model and date it was put into service.

**Figure 4:**
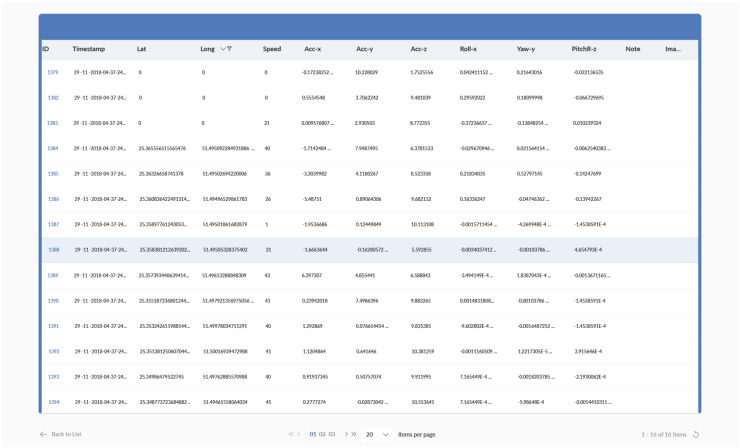
Screenshot of a real trip displaying the data recorded for every event.

**Figure 5:**
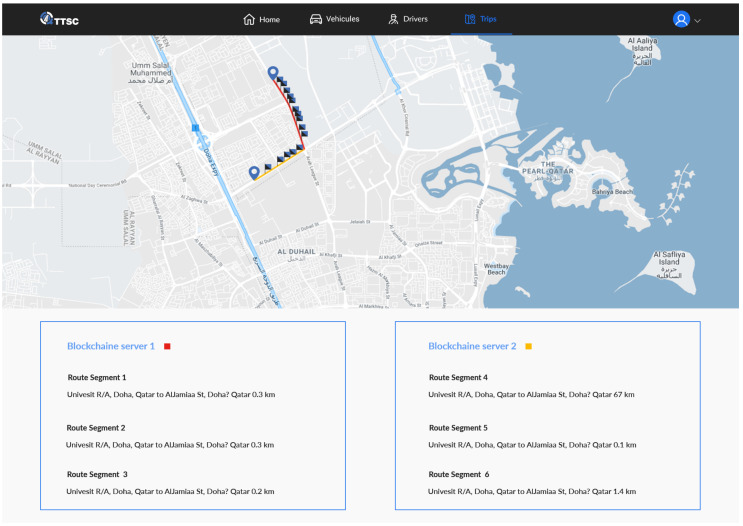
Screenshot of a real trip displaying the Blockchain layer for every road section.

By using Google Maps, the website displays the tracked trip and the position of individual events, as well as the details of all recorded events as shown in Figure 4 and Figure 5.

#### 4.4.2. The Blockchain Layer

##### Blockchain Layer Overview

**Figure 6:**
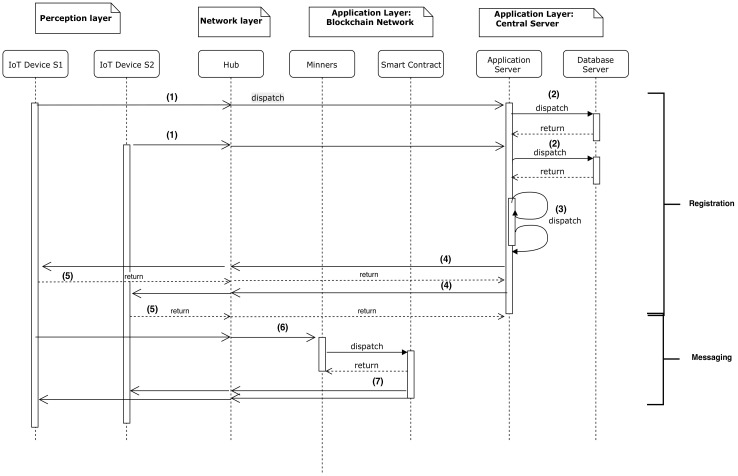
Sequence diagram of nominal scenario of communication between Internet of things (IoT) devices.

The Blockchain layer manages communication between cars. In each separate time slot, the car sends collected data to the central server via a web service. The data includes the current location and the status of the connection to one of the existing Blockchain layers. Subsequently, the central server invites nearby IoT devices to establish communication by an available Blockchain cloud. The communication is established after accepting the invitation. As illustrated in Figure 6, each road section contains a Blockchain layer, which sends the messages to the connected IoT devices.

##### System In- Depth

**Figure 7:**
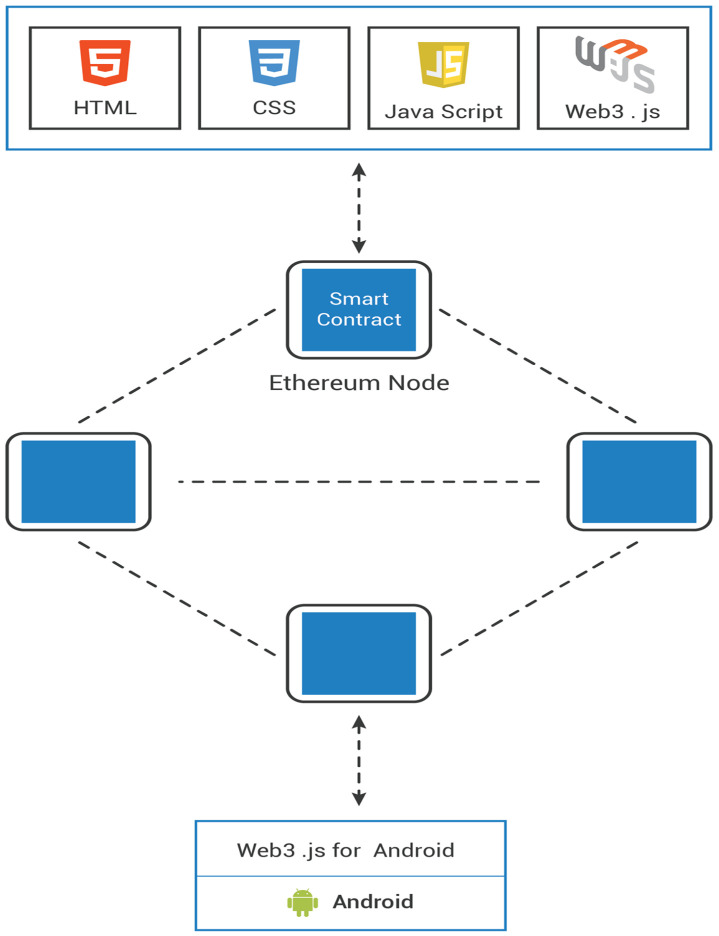
The architecture of decentralized applications (Dapp).

The Blockchain layer and the Android application jointly create decentralized applications (Dapp, dApp, or DApp). More precisely, decentralized applications represent distributed Internet apps run on a decentralized P2P network (Blockchain). Their code is an open source that is publicly open, and accordingly, it can be accessed and customized. Dapp applications do not rely on a central server like standard apps, as illustrated in Figure 7.

The Blockchain layer is the back-end of the decentralized applications, whereas the android application is the front-end part. The mobile app calls functions of the smart contract deployed in every Ethereum node in order to send a message via the Blockchain network. The communication passes through as a wrapper between the mobile and node-endpoint. This study uses one of the most reliable frameworks - the Web3.Js for Android framework. Its smart contract has two primary functions. The first function, setMessage, is in charge of publishing a new message on the Blockchain network in the amount of ETH the sender is willing to pay per unit of gas to mine the message. The second function, GetMessage, enables the device connected to the Blockchain network to read the existing data. Table 3 shows the format of the sent message. The message is composed of the following elements:

The message is composed of many elements:

- “s” field presents the information about the sender such as brand, car matriculation number, and color of the car.
- “ts” field defines the type of sender message that can be sent from a car, pedestrian or infrastructure.
- “t” field contains the time of sending the message. This helps the Android application to decide if the message is new or old. Therefore, if the mobile receives the message late, the notification alert will not be displayed.
- “tf” field contains the time when the message should be removed from the smart contract. Every time any participant adds a new message, it must delete the obsolete messages. Thus, the smart contract becomes lighter and remarkably reduces the execution time, mining time, cost and energy. The finish time value is the result of summing between the start time and duration of the message. The availability of the message in the smart contract is detailed in Table 4.
- “m” field includes the content of the message. For example : “Be careful of a drowsy driver, road crash in Khalifa Street”.
- “mt” field defines the priority of the message as explained in Table 5; there are three message types: information, warning, incident.
- “p” field contains the position of the sender and will not be displayed in case of delay in sending the message and when the sender becomes far from the incident point.

There are certain limits regarding the use of Blockchain for establishing communication between vehicles and other participants in the transportation system. The main concern is the time needed to update the transaction into a block chain. The DISV cannot be regarded as a real-time application due to the time needed for updating the smart contract. Therefore, several measures are proposed in order to reduce the content of the smart contract and consequently minimize the time required. First, instead of having one Blockchain layer in charge of the communication in a larger region, separate Blockchain layers within smaller areas would facilitate the communication of a low number of vehicles. Second, following the competition of their time, all messages should be removed from the system (see Table 4). Third, as elaborated in Table 3, the format of messages should be minimal to increase the transaction speed.

Furthermore, The Table 4 illustrates the duration of every message based on Urgent level, in fact the message is received by “SetMessage” function in smart contract will stored in Patricia Trie data storage on Ethereum Blockchain [48]. Once the become obsolete, The Smart contract remove the message after the central data receives a copy of it.

##### Nominal Scenario

In this section, the nominal scenario of communication between IoT devices is described. The sequence diagram in Figure 6 shows that the nominal scenario is divided into two sub-processes; registration and messaging. In the registration step, for every time slot (15 s), the IoT device sends the collected data to the central server via the Internet (1). The central server saves the collected data in the database server (2). In addition, the server looks for the IoT devices nearby places such as road section, roundabouts, traffic signals, or others (3). Subsequently, an invitation is sent to the devices in the same location to communicate through one of the available Blockchain layers (4). After accepting the invitation, (5) the second sub-process begins.

In the messaging step, the IoT devices are now connected to each other and can share data between them. The IoT devices send the message via the Blockchain network. Following the mining process (6), the message is added to the smart contract so that every device connected to this server Blockchain layer can receive it (7).

## 5. Performance Evaluation and Discussion

**Figure 8:**
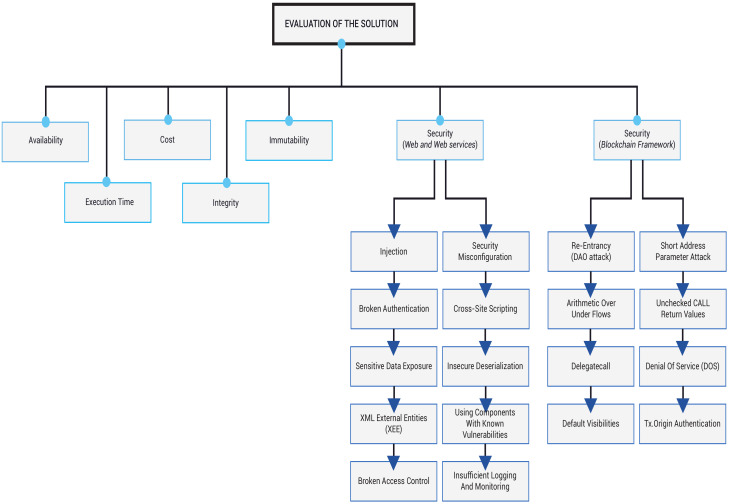
System evaluation diagram.

The performance of software solution can be assessed by applying different methodologies [49,50]. In particular, it is fundamental to evaluate the specific properties needed for the smooth functioning of the solution. The main properties of the proposed solution are execution time, costs, availability, integrity, immutability, and security as shown in Figure 8. All these properties must provide the highest performance in order to allow the flawless operations of the solution. Thus, this paper will address these properties to assess the overall performance of the solution.

### 5.1. Costs

The Testnet of the Ethereum network was used to deploy the prototype of the smart contract. In this section the costs of the creation and execution of the smart contract are analyzed. The following values, valid in January 2020 were used: 1 gas_1 wei (0.000000001 ETH) and 1 ETH $161,92 US. The minimum gas value to be used in a transaction was set at 1 wei; the average gas value was approximately 0.006845 Ethereum at the moment of analysis.

1Gas=0.006845Ethereum(ETH)

GasPrice=6,138,887Gwei

Table 6 presents the execution costs of various functions of the app. As summarized, the creation and deployment of the prototype on the Blockchain is the most expensive, $0.07572 US. However, it is worth mentioning that this is a one-time cost to set up and initialize the system. Moreover, this cost can be minimized by removing Truffle, which is the framework used for building, deploying, and managing smart contracts. It also includes features such as a “Migrations” contract to manage the deployment cycle.

The SetMessage function is not expensive, as it costs on average $0.03523 US. However, there are significant variations of the costs of “SetMessage” functions due to variable message input lengths. Nevertheless, the cost of one byte is set at 136 gas ($0.00003 US). In contrary, the GetMessage function does not require any additional cost because mining is not necessary while receiving messages from the blocks and no updates are needed for the smart contract.

### 5.2. Execution Time

One of the main criteria for evaluation of transportation management systems such as DISV is execution time. In fact, even a minor delay in sending or receiving messages can lead to severe disruptions of the system. For the proper functioning of the framework for communication between vehicles and all other actors in the transportation system based on blockchain technology, it is fundamental to ensure the timely addition of each message to the smart contract as the mining process relies on complex problem solving.

Considering that the proposed prototype is a real time application, execution time is very important. In computational tests, the call times for each function of the Android application are measured. In order to assess the performance of the Ethereum private Blockchain proposed solution, a server with a Configuration of 64 GB Ram and a Core i7-000 was used.

**Figure 9:**
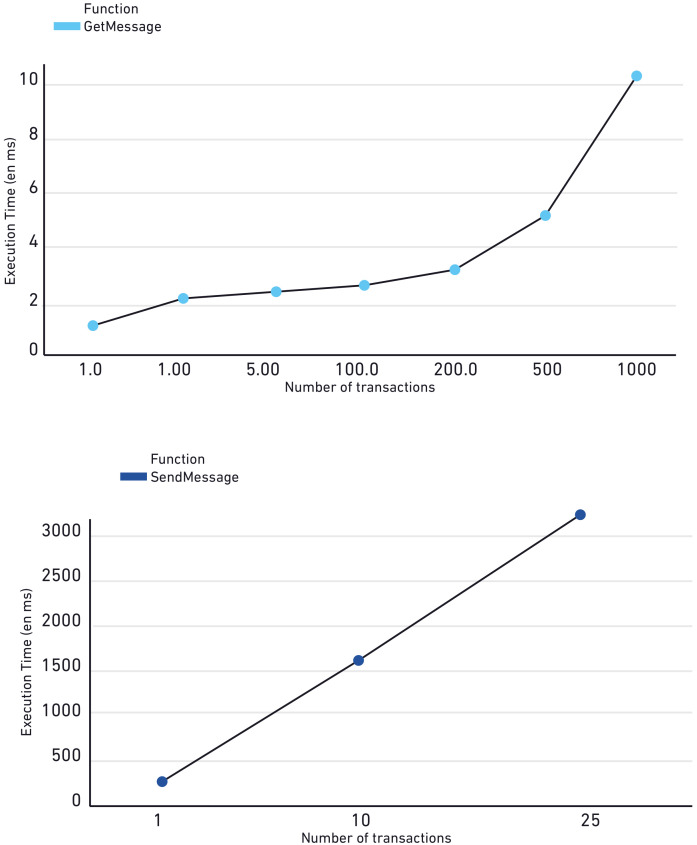
Execution time of the different functions in the Decentralized IoT solution for Vehicles communication (DISV) in milliseconds.

Figure 9 shows that the response time of the server of the GetMessage function is significantly shorter than the response time of the SetMessage.In fact, calling the GetMessage function needs between 1 milliseconds and up to almost 10 milliseconds when the server get 1000 requests. Therefore, there is no big difference in execution time between calling the GetMessage function one time or hundreds of times. However, calling the SetMessage function requires a lot of time because the message should be mined in order to add it into the smart contract. When the server receives ten requests of calling the SetMessage function, it takes 1.64 s and more than 90 s in the case of five hundred requests. Due to several factors, the computational study showed that is not recommended that the of list SetMessage exceeds 25 messages.

Real time execution is one major property of DISV since any transaction should take a very small running time. Therefore, it was recommended in the proposed architecture to use the Blockchain layer in every zone so the server receives and sends a small number of messages.

In addition to using many Blockchain layers, the Android application removes the old and duplicated messages so the smart contract contains only the necessary messages. Using the recommended architecture, in DISV, the response time of the messaging server is usually between 0 and 3 s. Hence, DISV can be considered a real-time application (RTA).

### 5.3. Memory and Power Consumption

**Figure 10:**
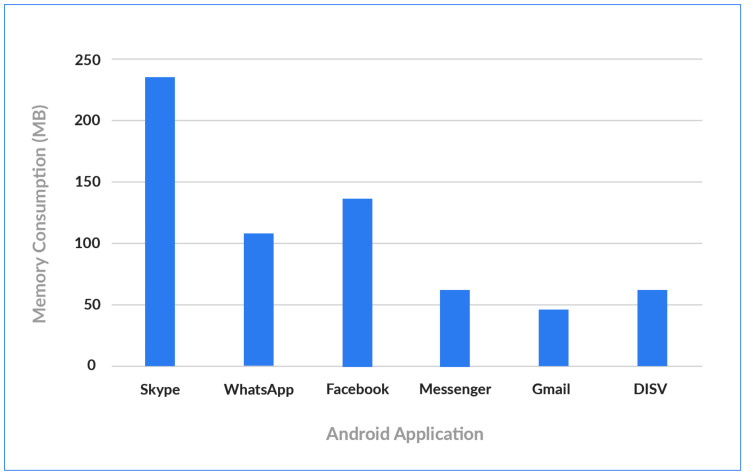
Comparing Memory Consumption of DISV with commercial mobile applications.

**Figure 11:**
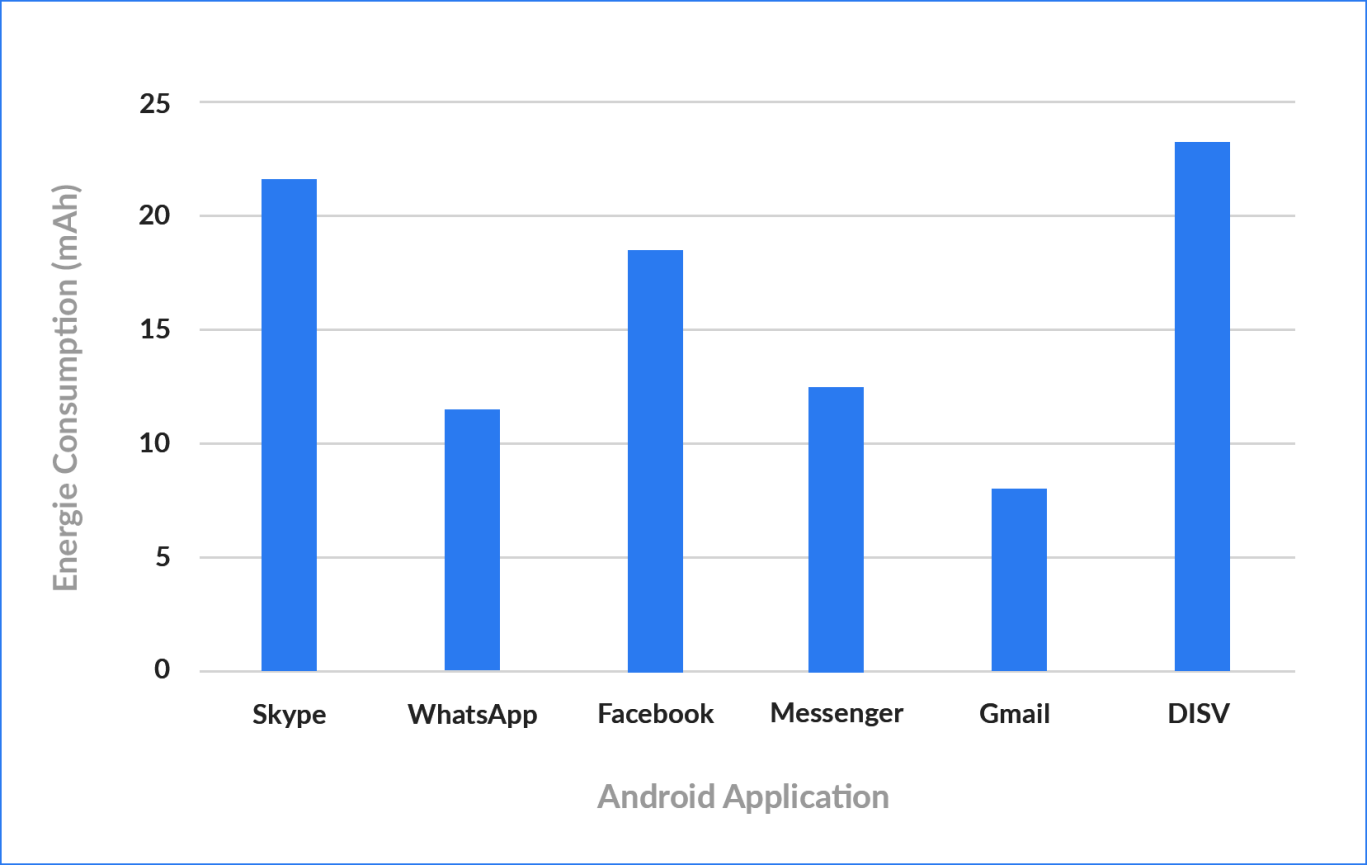
Comparing Energy Consumption of DISV with commercial mobile applications.

Since DISV uses Blockchain for the IoT, it is critical to evaluate Power and Memory consumption, considering that IoT devices usually have very low power and computational capacities. A Huawei P8 Lite with a Configuration of 2 GB Ram, Li-Po 2500 mAh battery and a Hisilicon Kirin 620 Processor was used in computation tests in the demo. Figure 10 demonstrates that the memory consumption of the developed Android solution is significantly lower than the consumption of other commercial applications, such us Facebook app (134 MB), WhatsApp (106), and Skype (233 MB). Regarding Energy Consumption, the proposed solution consumes electricity as average 23.43 mAh which is similar to Skype app and Facebook, which consume respectively 21.66 mAh and 18.56 mAh, as illustrated in Figure 11.

### 5.4. Availability

Another critical property of transportation management systems such as DISV is availability. More precisely, even the minor temporary shutdown of the system is likely to lead to traffic congestion and crashes. Availability means that a system is online and ready for access at any time. A variety of factors can cause a shutdown of the system (off-line), ranging from planned downtime for maintenance to sudden failure. The decentralized and robust nature of the Blockchain prevents attacks such as a denial-of-service (DoS) attack [51,52], which only target nodes [53], as the central party cannot be a single point of failure [52,54]. Yet, the distribution of the Blockchain is not complete. The mining power is typically limited to miners residing in approximately the same location. Thus, this enables isolating them by hijacking some border gateway protocol (BGP) prefixed with a routing attack employing the internet infrastructure [53]. Considering the comprehensiveness of the Internet, the Ethereum Blockchain network must be always reachable. Solutions that are centralized but whose databases are distributed are vulnerable to routing attacks because of potentially hindered communication with and between the physical databases. Thanks to the resiliency of the Blockchain malicious and damaged nodes on the network can be handled [55,56].

One of the greatest threats to the availability of a Blockchain solution is 51% attacks, which is the ability of someone controlling a majority of network hash rate to revise transaction history and prevent new transactions from confirming. As opposed to a public chain, these messages delays are combined with the heterogeneous power of miners in such a private chain could easily allow a 51% attack and lead to the Blockchain anomaly.

### 5.5. Integrity

Integrity is another fundamental property of the systems which exchange sensitive data among the users. Thus, it is necessary to assess the data integrity property of the proposed software solution. Data integrity refers to the accuracy and reliability of data through the whole life cycle. It is critically related to the concept of data security. Uncorrupted data is whole, and it remains unchanged in regard to its complete state. It is essential for ensuring security to keep data consistent throughout its life cycle. The reliability of data refers to compliance with the following standards:

- The accuracy of data—free from errors and confirmed by the protocol.
- The originality of data—accessible sources and preservation in the original form.
- Contemporary—data must be recorded at the exact time it was executed and observed.
- Legible—easy to understand, record permanently, and preserve original entries.
- An attributable—clear demonstration of who observed and recorded data, at what time, and what it is about.

Cryptographic Hashing and Merkle Trees are in charge of keeping the integrity of the data on public and private Blockchains intact. There are three main advantages of Merkle trees [56]. First, they ensure the validity and integrity of data. Second, their proofs are fast and computationally easy requiring less disk space or memory. Third, their management and proof needs minimal information to be transmitted across networks. Moreover, Cryptographic Hashing is critical for keeping the integrity and security of data recorded on Blockchain. Encryption guarantees security, whereas integrity is achieved by ensuring that signatures update when the data is changed. Therefore, considering that the DISV is based on blockchain technology, it ensures the communication between vehicles which maintains data integrity at all times.

### 5.6. Consistency

One of the major criteria for a new system evaluation is consistency which refers to the requirement that a series of measurements of the same project yields comparable results when different raters perform it by the same method. The proposed solution has employed the Ethereum Blockchain to build the consensus mechanism. Accordingly, as explained in [56], explicit reconciliation processes are not required. The consistency mechanism is based on the assumption that the branch behind the most Proof-of-Work represents the real branch. To ensure consistency, each block in the Blockchain accepted by a node preserves the consistency of the local replica of the database. In a case of a temporary disagreement among the nodes on the real consistent truths, Proof-of-Work enables the automatic resolving of the fork. Honest nodes cannot under any circumstances adapt to inconsistent chains. Within the network, the deeper buried blocks in the chain are always consistent. Considering Proof-of-Work prevents unsolvable reconciliation process, it is evident that the Blockchain ensures consistency in the proposed DISV system.

### 5.7. Confidentiality

Confidentiality in the context of computer systems allows authorized users to access sensitive and protected data. Thus, it is necessary to build in specific mechanisms to ensure confidentiality and safeguard data from harmful intruders. Confidentiality in a Blockchain setting allows involved parties to perform a transaction while preventing other participants from finding out certain facts or details about the transaction. Unlike public Blockchain such as Ethereum and Bitcoin that reject the concept of confidentiality and all their transactions are in the clear, confidentiality in private Blockchain can be handled if both the transaction and the identities of participating nodes are protected. To achieve these requirements, the proposed solution satisfies the following requirements:

- An unauthorized third party must be able to identify the counter-parties to a transaction in a Blockchain until the counter parties reveal that information.
- Transaction details must be invisible to the person who is not involved in that particular transaction until the participating parties don’t disclose their information.

### 5.8. Immutability

Immutability implies that alteration is not possible after the creation. To modify a transaction from history, it is necessary to re-mine all the blocks before the given block, which will reflect subsequently in each copy of the ledger in the network. It would also require rebuilding the Merkle tree of the block in which the transaction is located and redoing of all the proof of work for that block. Moreover, as the next block stores the hash of this block, it must also be re-mined. The subsequent block must be edited with the new “previous block hash”, which leads to a different block hash. Such a hash in certain cases would not match the set difficulty level, which implies re-mining of the block. In fact, the re-mining will have to occur until the final block in the chain. Simultaneously, while the miner re-mines old blocks, new blocks will be added to the chain. It means that in addition to re-mining the previous blocks to edit a historical record, the miner will have to edit newly generated blocks at the same time. This action is practically impossible due to the enormous computing power required. Accordingly, immutability is guaranteed in the proposed DISV system.

### 5.9. Security

Open Web Application Security Project (OWASP) Foundation’s [57] top web vulnerabilities was employed to assess the security of websites, web services, and the central server of the DISV system. OWASP is a non-profit organization whose mission is to offer practical and unbiased information on application security to security professionals and developers. It primarily focuses on the critical vulnerabilities of web apps. The vulnerability assessment analysis detected ten of the most common attacks presented in Table 7 together with security requirements needed to minimize vulnerability combat attacks. The recommended requirement is incorporated into the system. However, newly and complex attacks occur on a daily basis. Thus, applying the recommended prevention cannot entirely prevent the attacks, but rather minimize the possibility of damaging or hacking the system. The architecture and technology of Ethereum in regard to the smart contract have certain vulnerabilities, which are listed in Table 8.

Attacks on Ethereum are most commonly in relation to the external call. However, some attacks rely on particular functions and employ a loop in the smart contract.

The security of the proposed solution is considered high because it is using private Blockchain. In Table 8, several recommendations are presented.

## 6. Conclusions and Future Work

This paper introduced a new Decentralized IoT solution for Vehicles communication (DISV). It incorporates three primary layers to explore the possibility of using Blockchain for communication in the IoV. A prototype of the smart contract on the Testnet of Ethereum have been deployed. This study considered several properties of the solution, including availability, integrity, and security to test whether the Blockchain is an efficient and secure mechanism for IoV communications. The results showed that DISV can be considered as a real time application as well as a solution for the main challenges of Vehicle-to-X (V2X) communications such as security, centralization, and lack of privacy. In addition, it can facilitate the data exchange and cooperation between cars, infrastructure, and other actors of intelligent transportation systems. Moreover, DISV could be seen as an important element of the Advanced Driver Assistance Systems (ADAS) that might enhance the transportation safety and mobility. As a direction for future research, an enhanced version of DISV will be developed that includes reputation assessment system for unreliable data sources as in [58] Furthermore, a network for vehicle-based on Blockchain to allow users paying for electrical charging, parking spaces, and tolls through machine-to-machine transactions will be proposed.

## Figures and Tables

**Table 1:** Comparison between existing literature on the Blockchain solution for vehicle communication and the proposed system.

	Goals	Tuning Completeness	Performance
**M. Singh et al.**	Developing an Intelligent Vehicle-Trust Point protocol for secure and fast vehicular communications	No	No
**J. Kang et al.**	Developing a consortium blockchain-based distributed data management system	No	No
**B. et and al.**	Developing an Ethereum-based automatic management framework for distributed vehicular network	No	No
**E. Reilly et al.**	Developing a novel light client communications protocol based on Ethereum contracts for smart cities.	Yes	No
**G. Falco et al.**	Developing a Blockchain-based automotive hashing validation mechanism for vehicular data provenance	No	Yes
**S. Rowan et al.**	A novel and robust protocol for key production based on public-key Blockchain architecture with the use of both ultrasonic audio and visual light physical channels	No	No
**Proposed System**	Decentralized IoT Solution for Vehicles communication (DISV) based~ on the concept of Ethereum	Yes	Yes

**Table 2:** The main features of the developed IoT solution.

Layers	Developed Solution	Main Features
Perception layer	Android Application for Vehicles (AV)	Collects and analyze data about the trip, the vehicle, and the driver’s behavior.
	Android Application for Infrastructure (AP)	Simulate the role of IoT devices integrated into the roads such as radars, traffic lights, roadside electronic signs and other.
Network layer		Connects the sensors to other servers, networks devices and smart things.
Application layer	Blockchain Application	Managing communication between vehicles and other actors in the transportation system.
	Central Cloud Server	Processes and analysis obtained data Manages invitations of the of other actors.

**Table 3:** Model of the sent message in the Blockchain network.

Model	Example
{	{
Sender : ,	"s": "Toyota , 404551 , white",
TypeSender :	, "ts": "Car",
Time : ,	"t": " 2018-10-13 19:43:16",
FinishTime ,	"tf": " 2018-10-13 19:53:16",
Message ,	"m": "Alert Drowsy driver",
TypeMessage:	"tm": "3",
Position ,	"p": " 25.333091, 51.467223",
}	}

**Table 4:** Urgency level of the message sent through the Blockchain Network.

Urgent Level	Duration of Message	Example
0	10 min	Drowsy driver or bad driver behavior
1	1 h	Streets crowded
2	6 h	Temporary Closed Roads
3	12 h	Maintenance work

**Table 5:** Type of message sent through the Blockchain Network.

Type	Message
Information message	i.e., Informative messages from the Ministry of Interior. It is displayed only in the message page of the Android application.
Warning message	i.e., Information about traffic signal not working. It is displayed as a pop-up message.
Incident message	i.e., A drowsy driver, critical zone or extreme weather condition. It is displayed as a pop-up message with an alert sound to get the driver’s attention.

**Table 6:** Costs of the different functions in the Smart Contract based on 1 ETH = 161,92 USD and 1 gas = 0,000000001 ETH Rates.

Function	Gas Used	Price
Deploy Contract	389,473	0.07572 (one time/Truffle)
SetMessage	140,345–257,488	0.02486–0.0456
Get Message	0	0

**Table 7:** Open Web Application Security Project (OWASP) top web vulnerabilities and security and privacy requirements.

No.	System Attack	Description	Security and Privacy Requirements
A1	Injection	Injection Attacks (CRLF, LDAP, and SQL injection) take part when untrusted data is sent by an attacker to an interpreter and then executed as a command without adequate authorization.	The prevention is done by the system by ensuring that data is separate from queries and commands.
A2	Broken Authentication	Broken Authentication and Session Management vulnerabilities refer to users being able to work around sessions and authentic mechanisms or manipulate them.	The prevention is done by the system through strong storage mechanisms and password policies.
A3	Sensitive Data Exposure	Applications and APIs which do not have proper protection of sensitive data such as username and passwords and financial information. Accordingly, attackers can steal identities and commit fraud upon accessing such information.	It is necessary to have SSL incorporated into the system and to transfer sensitive data only with encryption such as AES-256. The detection of insecure obfuscation techniques is needed.
A4	XML External Entities (XEE)	Exploitation of vulnerable XML processors by attackers by uploading XL and inserting hostile content in an ML document. It can also be done by exploiting vulnerable integrations, dependencies, and code.	It is recommended to incorporate the Rest paradigm into the system and use data formats such as JSON. Also, avoiding the serialization of sensitive data is needed.
A5	Broken Access Control	This vulnerability occurs when users can access certain applications functionalities that are not intended for their use. Accordingly, they can modify a URL as a way to reach other functionalities.	It is necessary to incorporate a strong access control mechanism into the system.
A6	Security Misconfiguration	This is the case of insecure and outdated configurations and also not adequate protection of directories and files by a web server.	All components of an application, including an operating system, language runtime, and server must be suitably hardened following recommended best practices.
A7	Cross-Site Scripting	Attackers perform scripts using XSS in the victim’s web service endpoint or browser to redirect the user to malicious sites, deface websites orhijack user sessions.	To prevent malicious data from harming the database or website the system must render the correct data (Validate the Data).
A8	Insecure Deserialization	Insecure deserialization flaws allow an attacker to execute remote code in the application, elevate privileges, carry out injection and delete or tamper with serialized (written to disk) objects.	The system must incorporate SSL.
A9	Using Components With Known Vulnerabilities	Vulnerable components-frameworks, libraries, etc must run with full privilege.	The system must use approved enterprise libraries.
A10	Insufficient Logging And Monitoring	The detecting time of a breach is typically measured in weeks and sometimes months. Thus, in sufficient logging and ineffective integration, relevant security incident response systems allow attackers to reach other systems and become a persistent threat.	Monitoring systems such as Appdynamics and Dynatrace have defined rules and send proactive alerts. They should be incorporated into the system.

**Table 8:** Ethereum top web vulnerabilities and Security and privacy requirements.

No.	System Attack	Description	Security and Privacy Requirements
AE1	Re-Entrancy (DAO attack)	The smart contract of Ethereum can call and use codes of other external contacts, which can be hijacked and subsequently forced to conduct new codes through, for instance, a fallback function.	The transfer function should not send more than 2,300 gas with the external call, as it prevents the destination address/contract from calling another contract.
AE2	Arithmetic Over/Under Flows	The Ethereum Virtual Machine (EVM) determines fixed-size data types for integers. Attackers can exploit Variables in Solidity in a case of unchecked user input and performed calculations in numbers outside the range of the data type storing them them.	Protecting against under/overflow vulnerabilities is performed by designing or using mathematical libraries to replace the standard math operators, namely addition, subtraction and multiplication.
AE3	Delegatecall	Ethereum developers use the CALL and DELEGATECALL opcodes to modularize their code. However, DELEGATECALL can result in unintended code execution.	Solidity holds the library keyword to implement library contracts. (Check the Solidity Docs) Consequently, the library contract is non-self-destructable and stateless.
AE4	Default Visibilities	Solidity functions include visibility specifiers to dictate how functions are permitted to be called. Incorrect use of those specifiers results in serious vulnerabilities	The visibility of all functions should be specified in the contract.
AE5	Short Address/Parameter Attack	The parameters passed to the smart contract are encoded by the ABI specification. Sending encoded parameters shorter than the expected parameter length is possible.	Prior to sending the inputs to the Blockchain, they should be validated by the system.
midrule AE6	Unchecked CALL Return Values	Performing external calls in solidity can be done in several ways. Typically, the transfer method is used to send ETH to external accounts; while the send () function is employed for versatile external calls. Moreover, the CALL is used directly in solidity.	In all possible cases the transfer() function should be used instead of send() transfer() reverts when the external transaction reverts.
AE7	Denial of Service (DOS)	A DDoS attack on Ethereum Blockchain indicates that an attacker intends to use all resources of the network so that minors cannot record or cater to other transactions.	Contracts must not loop through data structures which allow artificial manipulation by external users.
AE8	Tx.Origin Authentication	Contracts authorizing users by the tx.origin variable are vulnerable to phishing attacks. These attacks trick users to carry out authenticated actions on the vulnerable contract.	Do not use tx.origin for authorization in smart contracts.
